# A case of schizophrenia refuting mind-body dualism

**DOI:** 10.4102/sajpsychiatry.v29i0.2081

**Published:** 2023-09-29

**Authors:** Mohlalefi C. Letuka, Tejil Morar

**Affiliations:** 1 Department of Psychiatry, Faculty of Health Sciences, University of the Witwatersrand, Johannesburg, South Africa; 2 Department of Psychiatry, Sterkfontein Hospital, Krugersdorp, South Africa

**Keywords:** psychiatry, mind-body dualism, duration of untreated psychosis, schizophrenia, holistic medicine, integrated care

## Abstract

**Introduction:**

The case report depicts the complex interplay between mental and physical illness and contests the notion of mind-body dualism in medicine. It emphasises the importance of holistic management of patients and the misnomer of schizophrenia as a purely mental illness.

**Patient presentation:**

Mr S is a 35-year-old male who presented to a South African specialist psychiatric hospital via the forensic system. He had multiple physical symptoms involving the abdominal, haematological, dermatological and neurological systems, in addition to an eight year duration of untreated psychosis with a marked decline in cognition and functioning.

**Management and outcome:**

An extensive medical examination during his admission excluded conditions such as early onset dementia, Huntington’s disease, pellagra, Wilson’s disease, autoimmune encephalitis and substance-related complications. A definitive diagnosis of schizophrenia was made, and both physical and psychiatric symptoms responded well to the administration of an antipsychotic resulting in an eventual discharge from the hospital.

**Conclusion:**

Mind-body dualism can result in a delayed diagnosis of schizophrenia and subsequent increased duration of untreated psychosis and other complications.

**Contribution:**

This case emphasises the flaws of mind-body dualism, and the interplay of mental and physical illness.

## Background

Attributed to Descartes in the 1600’s, mind-body dualism derived support largely as a model or belief system to explain illness at the time, rather than as a result of robust evidence.^1,2^ In a landmark article by Engel (1977), the theory of mind-body dualism was challenged and a new biopsychosocial model was proposed.^2^ However, the integration of physical and mental health has lagged.

Physical brain abnormalities have been observed in patients with mental illness, for example decreased cerebellar mass and neurological soft signs (NSS) in schizophrenia.^3,4^ Neurological soft signs are defined as subtle and non-localising neurological deficits in sensory integration, motor coordination, and sequencing of complex motor acts.^4^ Neurological soft signs are more prominent in patients with schizophrenia than the general population, even prior to initiation of treatment.^4^

In patients with schizophrenia specifically, a higher prevalence of human immunodeficiency virus (HIV), hepatitis, osteoporosis, altered pain sensitivity, sexual dysfunction, obstetric complications, cardiovascular diseases, obesity, diabetes, dental problems and polydipsia has been noticed compared with the general population.^5^ Patients with schizophrenia have a life expectancy that is 15–20 years less than the general population, with 60% of this mortality driven by comorbid physical illness.^2,5^

The case study that follows uniquely illustrates the clear overlap between mental and physical factors on a systemic and individual level. The patient presented to both general and psychiatric services and displayed a complex interplay of mental and physical issues on clinical presentation and one could not be separated from the other.

## Case presentation

Mr S is a 35-year-old single male with no children residing with his mother and siblings. His highest level of education is matric and he is unemployed. On admission he presented with auditory hallucinations and persecutory delusions since 2014 (see Table 1). He had become withdrawn and began wandering away from home for days at a time. A traditional healer was consulted, with no improvement. These symptoms were accompanied by a decline in functioning. Mr S had reportedly been social, friendly, able to sustain employment, manage finances, navigate to and from places by himself and had a good relationship with his brother prior to the onset of symptoms.

**TABLE 1:** Timeline of patient presentation.

Date	Event
2014	Onset of psychotic symptoms
August 2019	Charged with trespassing and taken into custody
September 2019	Transferred from prison to a tertiary academic hospital - admitted in the general medical ward and consulted by the psychiatry team. During this admission he was diagnosed with pulmonary tuberculosis and intellectual disability. Received treatment for tuberculosis but not initiated on any psychotropics.
November 2021	Complained of a painful rash on arms and legs in prison. Assessed as eczema with a superimposed infection and treated with flucloxacillin by a doctor in prison.
November 2021	Underwent 30-day forensic psychiatric observation, based on the psychiatric consultation in September 2019. Found unfit for trial and not responsible and placed on a waiting list for admission to a psychiatric hospital. Remained in prison for the interim.
January 2022	Referred from prison to a secondary level hospital and found to have iron deficiency anaemia. No features of upper gastrointestinal bleeding observed and no indication for gastroscopy documented. Discharged on lansoprazole and ferrous sulphate back to prison.
February 2022	Noticed to have maggots on his left leg in prison. Wound cleaned with normal saline and dressings applied. Patient was referred to a secondary hospital, treated with cloxacillin and discharged back to prison.
March 2022	Patient again referred to a secondary hospital after being observed to have poor hygiene and ongoing dry skin and rash in prison. Treated as an outpatient with antibacterial ointment, wound dressings, cloxacillin and analgesia.
March 2022	Admitted to a specialist psychiatric hospital from prison and initiated on an antipsychotic.
August 2022	Physical and psychiatric symptoms improved and patient discharged home.

Mr S was arrested in 2019 for a charge of trespassing and had no other forensic history. He was subsequently referred by court for a psychiatric forensic observation in October 2021. The assumption for the delay in his referral for forensic observation, is the long waiting list for observandi at state psychiatric facilities in South Africa. In October 2021, he underwent observation and was found unfit to stand trial and not criminally responsible. The differential diagnosis at the time was a major neurocognitive disorder and psychotic disorder. No psychotropics were initiated at the time and he was sent back to prison with the suggestion to be readmitted as an involuntary mental health care user for treatment. From prison, he was admitted to a psychiatric hospital for treatment in March 2022, after being on the waiting list for admission. This was his index presentation to psychiatry and he was antipsychotic naïve.

Mr S occasionally consumed alcohol. He did not meet the criteria for a substance use disorder on interview and this was confirmed by collateral. He had previously been diagnosed with pulmonary tuberculosis (TB) while in prison and completed six months of treatment (in February 2020). There is no family history of psychiatric or neurological illness.

Upon admission to the psychiatric hospital, Mr S was observed to be physically unwell. He presented with neurological signs (specifically cerebellar signs – ataxia, dysmetria dysdiadochokinesia), anaemia, a maculopapular rash, non-specific gastrointestinal symptoms (nausea, vomiting, abdominal pain and diarrhoea) and cachexia (body mass index 17.78). He scored 18 on the Scale for Assessment and Rating of Ataxia (SARA).

Upon a mental status examination, Mr S displayed a speech fluency disorder (stutter), psychomotor slowing, marked cognitive impairment, anosognosia and persecutory delusions. His mood was euthymic with a restricted affect and he displayed poor insight and impaired judgement. He scored 111 on the Positive and Negative Syndrome Scale (PANNS) and 12/30 on the Montreal Cognitive Assessment Test (MoCA).

## Investigations and differential diagnoses

An extensive medical work-up including infective markers, HIV, enzyme-linked immunoassay (ELISA) test, syphilis serology, thyroid function test, Vitamin B12, autoimmune markers, heavy metal studies, tumour markers, ceruloplasmin levels, lumbar puncture, a skin biopsy and electroencephalogram all came back normal. His computed tomography brain scan showed prominent sulci and gyri and cerebellar atrophy. His magnetic resonance imaging brain scan revealed age-inappropriate cortical and cerebellar atrophy. Mr S was consulted by a neurologist as well as neuropsychiatrist. Conditions such as early-onset dementia, Huntington’s disease, pellagra, Wilson’s disease, TB meningitis and autoimmune encephalitis were ruled out.

## Case management

A definitive diagnosis of schizophrenia was made. Mr S was initiated on risperidone 2 mg orally at night after a discussion with him as per National Institute of Clinical Excellence Guidelines,^6^ which was uptitrated to 4 mg during his admission. He was initiated on flupentixol decanoate 20 mg intramuscularly, monthly. He responded to antipsychotics and his PANNS score changed from 111 to 79 at discharge and a repeat MoCA had improved to 21/30. His cerebellar symptoms, cognitive symptoms and physical symptoms also improved on antipsychotics, with a SARA score of 5 at discharge. Mr S was discharged in August 2022.

## Discussion

Another consideration is the initiation of psychotropics indicated on observation of those who are found not fit and/or not responsible due to mental illness.^1,7^ A systematic review by Elyamani et al. (2020) highlighted the poor mental health literacy of non-psychiatrists in the ‘Arab Gulf countries’.^8^ In one of the studies included in the review, 54.3% had limited recognition of psychosis.^8^ In a study by Albin et al. (2020), 38% of psychotic participants were not diagnosed with psychosis at their initial contact with a clinician.^9^ These health system factors have been shown to contribute to the duration of untreated psychosis (DUP) in people with mental illness (PWMI).^10^ Duration of untreated psychosis is defined as the time from onset of overt positive psychotic symptoms to the initiation of antipsychotic medication.^10^ In a study by Oosthuizen et al. (2003), the mean DUP in a South African cohort was 229.10 ± 358.97 days.^11^ Research suggests that longer DUPs are associated with poorer treatment outcomes.^11^

Mr S had an approximately eight year DUP. During this time, he consulted a traditional healer and various medical doctors in prison and a tertiary medical hospital for physical complaints. Despite this, he was neither diagnosed with schizophrenia nor initiated on an antipsychotic. When he presented to a psychiatric hospital via the forensic system, he was physically ill. He had developed anaemia, cachexia and pulmonary TB likely secondary to the negative symptoms of schizophrenia. Had Mr S’s physical and mental health been treated holistically, he would have likely had a reduced DUP and subsequent improved outcome. Another consideration is the initiation of psychotropics on observandi that are found not fit and/or not responsible due to mental illness. Prompt initiation may reduce DUPs and improve outcomes for patients.

The case of Mr S illustrates the integration of physical and mental health. This case study emphasises the flaws of mind-body dualism and the misnomer of schizophrenia as a purely mental illness. The case also highlights the treatment of people with mental illness within the forensic system. Long waiting lists for observation, waiting lists for admission into psychiatric facilities and inadequate medical services in prison are all serious concerns, which need to be addressed.
